# Comprehensive Analysis of *NRG1* Common and Rare Variants in Hirschsprung Patients

**DOI:** 10.1371/journal.pone.0036524

**Published:** 2012-05-04

**Authors:** Berta Luzón-Toro, Ana Torroglosa, Rocío Núñez-Torres, María Valle Enguix-Riego, Raquel María Fernández, Juan Carlos de Agustín, Guillermo Antiñolo, Salud Borrego

**Affiliations:** 1 Department of Genetics, Reproduction and Fetal Medicine. Institute of Biomedicine of Seville (IBIS), University Hospital Virgen del Rocío/CSIC/University of Seville, Seville, Spain; 2 Centre for Biomedical Network Research on Rare Diseases (CIBERER), Seville, Spain; 3 Department of Pediatric Surgery, University Hospital Virgen del Rocío, Seville, Spain; University of Hong Kong, Hong Kong

## Abstract

Hirschsprung disease (HSCR, OMIM 142623) is a developmental disorder characterized by the absence of ganglion cells along variable lengths of the distal gastrointestinal tract, which results in tonic contraction of the aganglionic gut segment and functional intestinal obstruction. The *RET* proto-oncogene is the major gene for HSCR with differential contributions of its rare and common, coding and noncoding mutations to the multifactorial nature of this pathology. Many other genes have been described to be associated with the pathology, as *NRG1* gene (8p12), encoding neuregulin 1, which is implicated in the development of the enteric nervous system (ENS), and seems to contribute by both common and rare variants. Here we present the results of a comprehensive analysis of the *NRG1* gene in the context of the disease in a series of 207 Spanish HSCR patients, by both mutational screening of its coding sequence and evaluation of 3 common tag SNPs as low penetrance susceptibility factors, finding some potentially damaging variants which we have functionally characterized. All of them were found to be associated with a significant reduction of the normal NRG1 protein levels. The fact that those mutations analyzed alter NRG1 protein would suggest that they would be related with HSCR disease not only in Chinese but also in a Caucasian population, which reinforces the implication of *NRG1* gene in this pathology.

## Introduction

Hirschsprung disease (HSCR, OMIM 142623), a developmental disorder occurring in 1 of 5,000 live births, is characterized by the absence of ganglion cells along variable lengths of the distal gastrointestinal tract, which results in tonic contraction of the aganglionic gut segment and functional intestinal obstruction. Such aganglionosis is attributed to a failure of neural crest cells to migrate, proliferate, and/or differentiate during enteric nervous system (ENS) development in the embryonic stage [1], [2]. HSCR most commonly presents as isolated cases and displays a complex pattern of inheritance with low, sex dependent penetrance and variable expression.

The *RET* proto-oncogene (OMIM 164761) is the major gene associated to HSCR with differential contributions of its rare and common, coding and noncoding mutations to the multifactorial nature of this pathology [3], [4]. In addition, numerous molecular genetic studies have identified rare coding mutations in many other genes (*GDNF, NRTN, PSPN, EDNRB, EDN3, ECE1, NTF3, NTRK3, SOX10, PHOX2B, L1CAM, ZFHX1B, KIAA1279, TCF4, PROK1, PROKR1, PROKR2* and *NRG1*) related to HSCR [2], [3], [5]–[9]. However, cumulatively, the conventional mutations related to HSCR reported so far explain less than 5% of cases, being the vast majority of them long segment HSCR/total colonic aganglionosis (L-HSCR/TCA) and syndromic forms of the disease.

HSCR is regarded as a complex and multifactorial disorder, in which the contribution of several different loci acting in an additive or multiplicative manner is usually required to cause the disease [3]. Because of this evidence, many different techniques have emerged to identify new HSCR susceptibility loci, such as genome wide linkage and genome wide association studies (GWLS and GWAS respectively) [10], [11]. In this way, several HSCR-associated regions, such as 16q23 [10], 21q21 [11], 9q31 [12], 19q12 [13], 3p21 [13], [14] or 4q31.3-q32.3 [15] have been described, although the genes underlying such associations have not been identified yet in the majority of the cases.

A recent GWAS has successfully identified *NRG1* as a new candidate gene for HSCR [16]. To refine the locus on 8p12 that had resulted to be linked [16], a total of 243 SNPs were genotyped in Chinese HSCR patients and controls [17]. Genotype analysis narrowed down the HSCR-associated region to six of the most associated SNPs (rs16879552, rs7835688, rs10088313, rs10094655, rs4624987 and rs3884552) mapping to the *NRG1* promoter. Moreover, significant differences in *NRG1* expression levels between patients and controls bearing the same rs10088313 risk genotype were detected [17]. This seems to indicate that the effects of *NRG1* common variants are likely to depend on other alleles or epigenetic factors present in patients and would account among other factors for the variability in the genetic predisposition to HSCR. Finally, the implication of *NRG1* in HSCR has been demonstrated through the identification of coding mutations whose pathogenic role was demonstrated by different functional approaches [8]. In such study, the authors also reported the expression of *NRG1* and its receptors in gut, although only *NRG1* type I HRG-ß1 (NM_013956.2) was detected in HSCR and control guts but no isoforms type II or III.

A recent study, using TaqMan single-nucleotide polymorphism genotyping and PCR-restriction fragment length polymorphism, to validate the association of the *RET*-protooncogene and the *NRG1* in HSCR in Thai sporadic HSCR cases, has been reported. The rs2435357 (RET-protooncogene) and rs2439305 (NRG1) showed the strongest associations with the disease. They concluded that the genetic variation of the *RET*-protooncogene and *NRG1* is involved in the risk of HSCR development in the Thai population. Moreover, the study also detected a combined effect of SNPs by SNP-SNP interaction, which may help in predicting HSCR risk [18].

NRG1 is a trophic factor that contains an epidermal growth factor (EGF)-like domain that signals by stimulating ErbB receptor tyrosine kinases. Most NRG1 isoforms are synthesized as membrane-anchored precursors called pro-NRG1s that are subsequently, cleavaged to be released into the extracellular medium, where they can act by activating ErbB-mediated pathways. The NRG1 type I isoforms present a single hydrophobic sequence that crosses the plasma membrane, leaving exposed on the surface the EGF module and the other domains that are in N-terminal position, as immunoglobulin (Ig-like) domain [19], [20], [21], [22]. This area is flanked by basic amino acid sequences which could act as anchor sequences and control that the topology of the molecule is correct [21]. It has been proposed that the transmembrane sequence of the NRG1 could act as a signal peptide controlling its association with membranes and its entry into the signalling pathway since, as mentioned above, these molecules have a consensus sequence at its N-terminal to carry out this function [23], [24]. The signal triggered by the union of NRG proteins with their ErbB receptors affect some cellular processes as proliferation, differentiation, migration, apoptosis and cellular survival [25]–[27]. It has been previously described that NRG1 receptors ErbB2/ErbB3 are expressed in mouse vagal neural crest cells entering the developing gut and in adult intestinal epithelia of both humans and mice [28]–[31]. In addition, *NRG1* is also expressed in mice and human intestinal mucosa and enteric ganglia [32], [33]. The loss of ErbB2 signalling in the colonic epithelial cells in mice led to postnatal colonic aganglionosis because in absence of that receptor the production of survival factors required for the postnatal maintenance of the ENS could not be induced by NRG1 [34].

The genetic and functional evidence for a role of *NRG1* in ENS and more specifically in HSCR [8], [29], [35], led us to perform a complete evaluation of this gene in the context of this disease in a series of Spanish patients.

## Materials and Methods

### Patients and Controls Subjects

In this study we have included a total of 207 Spanish HSCR patients (23% female, 77% male), and their parents when available. 188 were sporadic cases, while 19 were familial cases belonging to 13 different families. In addition, we also analyzed a group of 150 normal controls comprising unselected, unrelated, race, age, and sex-matched individuals.

A written informed consent was obtained from all the participants for clinical and molecular genetic studies. The study was approved by the Ethics Committee for clinical research in the University Hospital Virgen del Rocío (Seville, Spain) and complies with the tenets of the declaration of Helsinki.

### Mutational Analysis

Genomic DNA was extracted from peripheral blood leukocytes from all the individuals included in the study, using standard protocols.

The mutational screening of the complete coding sequence of *NRG1* (HRGß1 isoform) was carried out by denaturing high-performance liquid chromatography in a WAVE DNA Fragment Analysis system (Transgenomic). Those fragments with aberrant profiles were subjected to sequence analysis (NRG1 NM_013956.3) using an ABI Prism®3730 Genetic Analyzer and the SeqScape® v2.5 software (Applied Biosystems).

When a novel change was detected, the appropriate DNA fragment was also screened in a group of 150 normal controls, in order to determine if the variant was a common polymorphism. Moreover, parental DNA, when available, was used to perform the segregation analysis of the identified variants.

To predict the putative pathogenic role of the novel variants at the protein sequence level, we selected the SIFT and Polyphen tools (http://blocks.fhcrc.org/sift/SIFT.html; http://genetics.bwh.harvard.edu/pph/).

Novel variants located within the non-coding region were submitted to several Splice Sites and Transcription Factors Binding sequences prediction interfaces such as http://www.fruitfly.org/seq_tools/splice; html; http://www.fruitfly.org/cgi-bin/seq_tools/promoter.pl and http://www.ebi.ac.uk/asd-srv/wb.cgi).

The NRG1 protein sequences were submitted to ScanProsite (http://expasy.org/tools/scanprosite/) to scan for the occurrence of patterns, profiles and motifs stored in the PROSITE database.

### Large Scale Genotyping

Three tag SNPs were analyzed in our 134 HSCR trios and controls using Taqman® technology, in a 7500 Fast Real-Time PCR System (Applied Biosystems) (conditions available upon request). They had been previously described as associated with HSCR [rs16879552 C>T; rs7835688 C>G and rs10088313 G>T] in the Chinese population [16], [17]. For each polymorphism, deviation of the genotype frequencies from those expected under Hardy-Weinberg equilibrium was assessed. Allelic and genotypic frequency and distribution of the three polymorphisms were calculated and then compared between patients and controls. Moreover, parental haplotypes were constructed as previously described [36] in the context of the affected children’s haplotypes, so that transmitted and non-transmitted haplotypes from unaffected parents to affected children were noted and their frequencies compared.

### Generation of Mutated Constructs

Full-length human *NRG1* cDNA (NRG-1 NM_013956.3) subcloned into a pFLAG-CMV expression vector was provided by Garcia-Barcelo MM (Hong Kong, China). The mutations M139I, M111T and R438H were generated through PCR-mediated site-directed mutagenesis (Quick-Change XL site-directed mutagenesis kit (Stratagene) and confirmed by direct sequencing.

### Cell Culture and Transient Transfection

COS-7 cells were obtained from ATCC (CRL-1651) and maintained in DMEM medium containing 10% FBS, penicillin/streptomycin and L-glutamine (Invitrogen).

For expression studies by immunoblotting, 8 µg of plasmids carrying wild-type (wt) or mutant (mt) NRG-1 cDNAs were transiently transfected into 4×10^5^ COS-7 cells (100 mm dishes) using FuGene (Promega) following manufacturer’s instructions. Cells and conditioned medium were collected 24 hours after transient transfection and used for the subsequent experiments. We obtained a transfection efficiency around 80%.

### Western Blotting

Cells from three 100 mm dishes were collected and then lysed with cell lysis buffer (20 mM TrisHCl pH 8.0, 135 mM NaCl, 1 mM EDTA, 1.5 mM MgCl_2_, 1% Triton X-100, 10% glycerol and a cocktail of proteases inhibitors). In addition, conditioned medium was collected, filtered through 0.2 µm pore size sterile filter unit (Millipore) and then concentrated up to 30 folds by centrifugation through an Amicon-10 K (Millipore) concentrator. Protein concentrations were estimated using the Bradford (Bio-Rad) assay with bovine serum albumin as a standard.

Thirty µg of proteins obtained from cell extracts and 100 µg from conditioned media were separated by SDS–PAGE on 10% polyacrilamide gels, transferred onto polyvinylidene difluoride membranes (Hybond, GE Healthcare) and probed with primary antibodies overnight at 4°C. A rabbit polyclonal anti-NRG1 (1∶2000; F20, Santa Cruz Biotechnology) and a rabbit polyclonal anti-FLAG M2 (1∶10000, Sigma Aldrich) were used as primary antibodies.

Membranes were washed and incubated with secondary antibody anti-rabbit HRP-conjugated (1∶7000; Cell Signalling) for 90 min at room temperature, followed by detection using ECL reagents (GE Healthcare). The same membranes were stripped with stripping buffer (100 mM 2-mercaptoethanol, 2% sodium dodecyl sulfate, 62.5 mM Tris–HCl, pH 6.7), blocked and reprobed with monoclonal antibody anti-α-tubulin (1∶5000; Sigma Aldrich) to ensure equal loading of cell protein per lane.

The representative pictures of at least three independent assays were analyzed. The bands were scanned and quantified by Quantity One software (Bio-Rad). The results were corrected for background values and normalized and expressed relative to the internal control, α-tubulin. Data were analyzed using the paired Student’s t test. A p value<0.05 was interpreted to represent a statistically significant difference.

### Immunocytochemistry

Twenty-four hours after the transient transfection with 2 µg each DNA onto 6-well plates, the transfected cells were fixed with 4% paraformaldehyde. Expression of the protein was monitored by immunostaining with rabbit polyclonal anti-NRG-1 (F20, 1∶5,000, Sigma-Aldrich). After washing, the immunosignals were then detected using the secondary antibody conjugated with Texas red (1∶200; Alexa 568, Invitrogen). All cultures were counterstained with DAPI to detect nuclei. Cells were photographed using an Olympus BX61 microscope with a digital camera DP72 under fluorescence illumination (Olympus España, S.A.U.).

## Results

### Analysis of NRG1 Variants and Haplotypes

We conducted a case-control association study to discern whether any of the variants were directly related to the HSCR phenotype in our population. Allelic and genotypic frequencies and distribution of the *NRG1* polymorphisms showed no statistically significant differences in patients *versus* controls (Table 1). Similarly, no significant differences were obtained in the comparison of transmitted *versus* non transmitted alleles or haplotypes of *NRG1* from healthy parents to their affected children (data not shown).

**Table 1:** Allelic distribution and frequency of *NRG1* genotyped variants.

Variant	Allele	HSCR (%)	Controls (%)
rs16879552 C>T	C	267 (99.6%)	262 (97.8%)
	T	1 (0.4%)	6 (2.2%)
		**γ^2^ = 2.32, p = 0.123**	
rs7835688 C>G	C	131 (48.8%)	114 (42.5%)
	G	137 (51.2%)	154 (57.5%)
		**Γ^2^ = 2.17, p = 0.140**	
rs10088313 G>T	G	265 (98.9%)	263 (98.1%)
	T	3 (1.1%)	5 (1.9%)
		**γ^2^ = 0.13, p = 0.725**	

Allelic distribution and frequency of the *NRG1* genotyped variants in HSCR patients and controls and their statistical comparison through **γ** ^2^.

### Mutational Analysis

The screening of the coding regions of *NRG1* (HRGß1) gene in our cohort of 207 Spanish HSCR patients revealed 18 variants (Table 2), nine of them were located within the non-coding region. After the bioinformatic predictions, we failed to find that any of those variants would affect neither the splicing process nor the formation or modification of a transcription factor binding site in the DNA sequence.

**Table 2:** Sequence variants detected in our cohort of HSCR patients.

Nucleotide Change	Amino acid Change	Reference
c.332T>C	p.M111T	–
c.417G>A	p.M139I	–
c.1313G>A	p.R438H	–
c.-97 C>A	–	rs7834206
c.-266 T>C	–	rs7820838
c.101–56_68del	–	–
c.113G>A	p.R38Q	rs3924999
c.229T>C	p.L77L	–
c.400+5 G>C	–	–
c.401−50C>G	–	–
c.414C>T	p.G138G	rs79277882
c.502+31230G>C	–	rs35641374
c.502+31212G>C	–	rs34822181
c.691+553A>G	–	rs79916768
c.796G>T	p.V266L	rs74942016
c.881T>C	p.M294T	rs10503929
c.819–26delA	–	rs67120632
c.1648C>T	p.R550W	rs80127039

Compilation of all the sequence variants obtained through our genetic analyses.

Regarding the coding region, three out of the remaining nine variants (M111T, M139I, R438H) were novel variants and were absent in the 150 controls tested (Table 3). We checked the 1000 Genomes Project, the NCBI and dbSNP databases and Ensembl for the presence of the variants identified in our HSCR patients and none were previously described.

**Table 3:** Candidate variants detected in HSCR patients.

Nucleotide change	Amino Acid change	In silico prediction	LS	Inheritance	Other
c.332T>C	p.M111T	Probably damaging	S-HSCR	Father	*RET*: A373V
			S-HSCR	Father	*RET*: A373V
			S-HSCR	Mother	–
c.417G>A	p.M139I	Benign	S-HSCR	*De novo*	*–*
c.1313G>A	p.R438H	Probably damaging	S-HSCR	NA	–
			S-HSCR	NA	–

List of details of the candidate variants in *NRG1* isoform ß1 to be mutations associated with HSCR phenotype analyzed.

* NA: Not available.

LS = Length segment.

Other = Mutations in other genes.

It is worthy to mention that the M111T variant was present in three patients and two of them also harbour the A373V *RET* mutation previously associated with HSCR. In addition, M139I was a *de novo* variant present only in one patient (Table 2).

**Figure 1:**
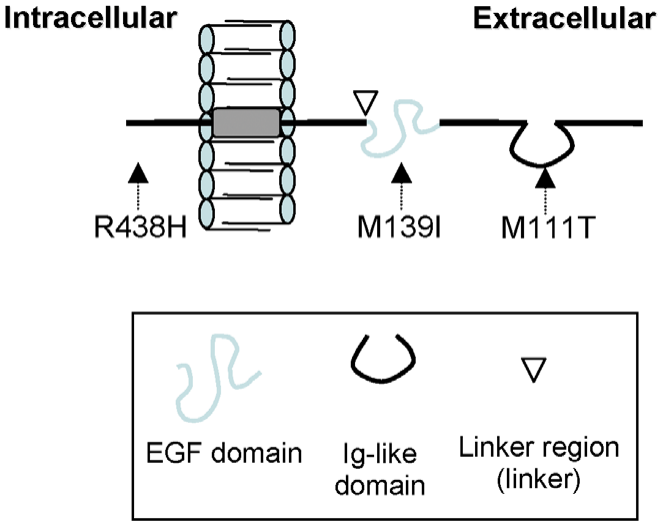
Mutant NRG1 proteins. Scheme of the main regions of NRG1 isoform ß1 protein and the location of the three variants analyzed.

Based on bioinformatic predictions (http://prosite.expasy.org/; http://emboss.bioinformatics.nl/cgi-bin/emboss/garnier), none of the three variants studied affected the transmembrane domain, although all of them affected functional domains of the wild-type protein (Figure 1). In addition, based on Polyphen and SIFT tools as indicators of functional relevance, M111T and R438H were predicted to be probably damaging. Although M139I was predicted to be benign, it was a *de novo* variant and therefore we perform to *in vitro* methods to further discern pathogenic effect in these three new variants (Table 2).

The location of the affected residues in the NRG1 protein (M111T in the Ig-like domain, M139I flanking the EGF-like domain and R438H in the cytoplasmic domain) (Figure 1) suggests that M111T may affect the stability of the protein on the extracellular domain and that M139I could affect the receptor activation of the NRG1 protein. As well as our R438H mutant protein, it has been previously reported that a NRG1 variant located at the cytoplasmic domain, would affect the proteolytic cleavage of the pro-NRG1 protein [8].

Therefore, we decided to analyze if any of M111T, M139I and R438H variants would affect the quantity of NRG1 normal protein both at total cell lysates and at concentrated conditioned media based on the type of processing of this type of proteins.

**Figure 2:**
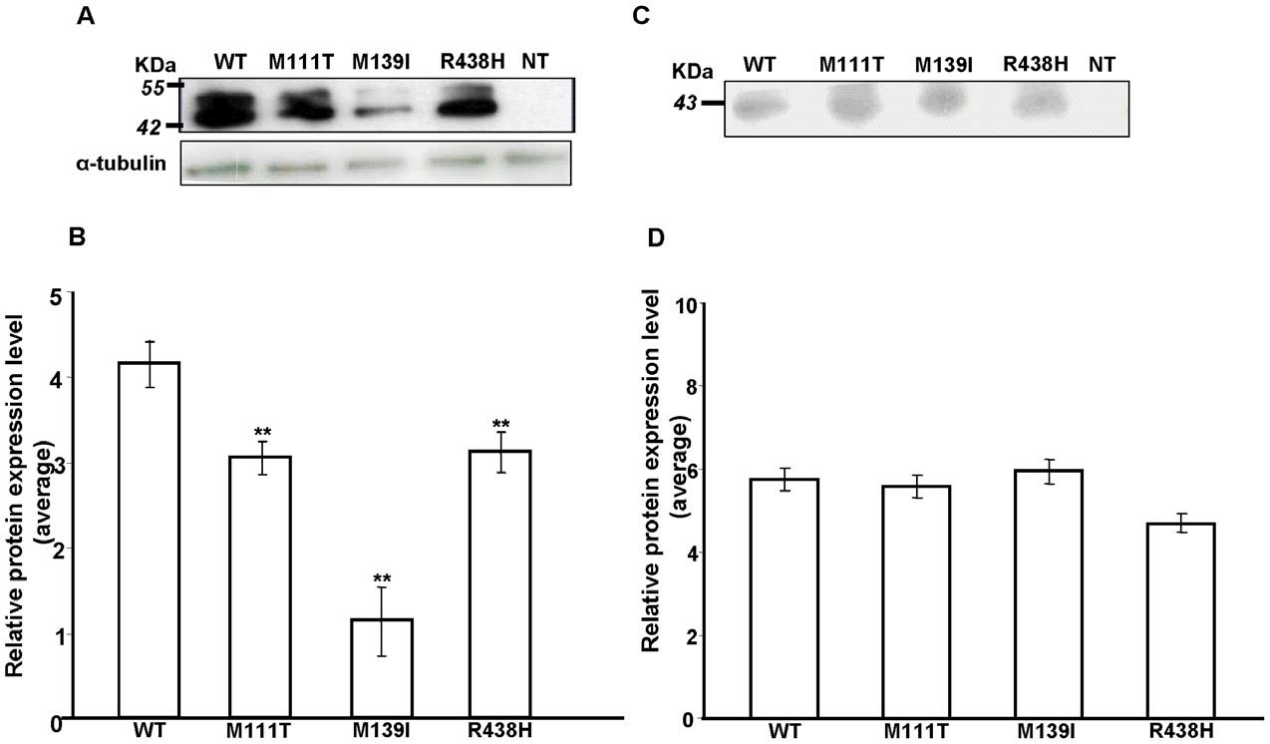
Immunoblot analysis of NRG1 mutants. Human NRG1 wildtype (WT) and mutants (M111T, M139I and R438H) were overexpressed in COS7 cell line. A) The intracellular levels of NRG1 wild-type and M111T, M139I and R438H mutants were detected with anti-neuregulin 1α/ß1/2 (F-20) antibody. All three mutants showed a significant lower protein expression. Alpha-tubulin was used as the loading control for normalization. B) The *bar chart* represents the quantitative data of the relative protein expression levels normalized with alpha-tubulin in three independent assays. (**p<0.005). C) The release of the extracellular domain in the medium was detected using anti-FLAG M2 antibody. The mutations did not affect the release of that domain of the NRG-1 protein. D) The *bar chart* represents the quantitative data of the relative protein expression levels normalized in three independent assays.

Both wt and mt proteins were generated to subsequently study the levels and cellular location of the NRG1 proteins. Immunoblotting assays performed with whole cell lysates showed a significant reduction (M111T p = 0,0019; M139I p = 0,0012; R438H p = 0,0026) on the total quantity of the three mutant proteins (M111T, M139I and R438H) in comparison with the wt protein (Figure 2A and 2B). Reduced amount of these NRG1 mutant proteins would suggest alteration of the signalling pathways regulated by NRG1, although further experiments are required to discern this hypothesis. In order to check the modifications on the cleavage processes for the variants tested, we have performed immunoblotting with conditioned media of each sample but any significant alteration was detected (Figure 2C and 2D).

**Figure 3:**
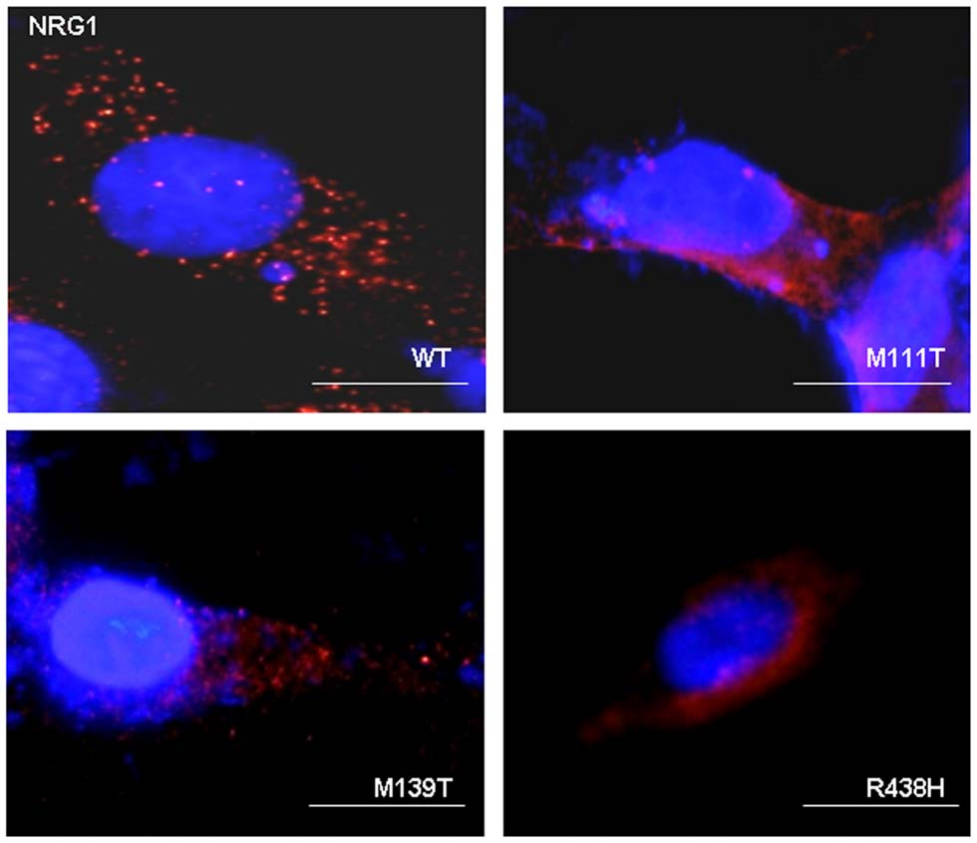
Analysis of cellular localization of NRG1 mutants. Human NRG1 wildtype (WT) and mutants (M111T, M139I and R438H) were overexpressed in COS7 cell line. Cellular localization of NRG1 wild-type and mutants were analyzed using immunostaining with anti-neuregulin 1α/ß1/2 (F-20) antibody. Immunosignal for the three mutants was different in comparison with WT. Patchier and perinuclear distribution was observed in the three mutants in comparison with the punctuate at cytoplasmic membrane distribution of the WT protein.

Immunocytochemistry revealed a different distribution of the wt and mt NRG1 proteins in the cytoplasmic organelles, suggesting that all three mt affect the NRG1 protein distribution or location. We could observe a typical punctuate pattern for the wt protein, corresponding to a protein anchored to the cytoplasmic membrane, although the three mt proteins showed a patchier and perinuclear distribution, which showed a different distribution in comparison with the wt protein (Figure 3).

## Discussion

In the present study we have performed a mutational screening and analysis of common sequence variants on the *NRG1* gene in a Spanish cohort of HSCR patients.

We did not find any significant difference after comparing allelic and genotypic frequencies of the *NRG1* polymorphisms analysed in HSCR vs controls. These results are not concordant with those previously described in the Chinese population, in which a clear over-representation of the three variants was found in the HSCR series, suggesting that the association of such polymorphisms to the disease is restricted to such specific population. In fact the existence of other genetic factors conferring susceptibility to HSCR in specific populations has been repeatedly reported. For instance, it has been reported that there exist two different *RET* haplotypes encompassing the enhancer mutation that are over-transmitted to the HSCR offspring in Caucasian populations (Spanish, Italian, French, and Dutch), while in the Chinese sample only one of those haplotypes was present [3]. A possible explanation was that the enhancer mutation arose on one haplotype which, after the Asian-European split, rearranged to give also the other haplotype, but exclusively in the European part. A similar hypothesis could be forwarded to explain the *NRG1* effects in the Chinese population, being this supported by the fact that great differences are observed among the frequencies of the studied variants between both populations (rs16879552 Spanish = 0,4%–2,2% vs Chinese = 38%–51%; rs 7835688 Spanish = 51,2%–57,5% vs Chinese = 15%–26%; rs 10088313 Spanish = 1,1%–1,9% vs Chinese = 40–52%). That difference among Caucasian and Asian populations had been recently corroborated by a recent genotyping study in Thai population, where the genetic variation of the *RET*-protooncogene and *NRG1* is involved in the risk of HSCR development in the Thai population [18].

Previous studies had indicated that NRG1 is a signalling protein that mediates cell-cell interactions and it is essential for the development and function of multiple organ systems and its dysregulation has been linked to diseases such as breast cancer, schizophrenia and HSCR [8], [29], [37]. In addition, it has been shown that not only common, but also rare variants of the *NRG1* gene contribute to HSCR [8]. Here we report some novel variants located within the non-coding region although after bioinformatic predictions we failed to find that any of those variants would affect neither the splicing process nor the formation or modification of a transcription factor binding site in the DNA sequence. Furthermore, we report three new missense mutations (M111T, M139I and R438H) as probably causing mutations for HSCR. Those variants were located on functional domains within the protein and all of them were found totally absent in control population. After functional approaches, we found that M111T, R438H and M139I mutant proteins induced a significant reduction in the quantity of the normal NRG1 protein levels in cells expressing them. In fact, our *in silico* predictions revealed that M111T and R438H would be probably damaging. Two of the affected residues in the NRG1 protein were located at the extracellular domain (M111T was in the Ig-like domain and M139I was in the EGF domain) and the other one, R438H, was located at the cytoplasmic domain. The Ig-like domain in NRGs proteins could act in the process of attenuation of signalling through ErbB receptors, promoting the internalization and degradation of the complex ligand-receptor. This could be a control mechanism of the NRGs biological activity limiting their ability to diffuse freely and allowing the intracellular accumulation of these proteins to act quickly after being processed. The variant M111T detected at this domain would influence this process of attenuation which would explain the significant reduction of NRG1 level detected in cell lysates and the absence of differences obtained in the conditioned media. In addition, there are two important facts to mention about M111T variant: First, the change of a methionine by a threonine means that it is a non-conservative mutation. This aminoacidic change would alter structures and/or functions of NRG1 protein, as we can guess by functional approaches. Secondly, the A373V *RET* mutation was also present in two out of three patients with this *NRG1* mutation, both with paternal inheritance. Both patients have the A373V *RET* mutation with maternal inheritance. The *NRG1* and *RET* loci would act in an additive manner, which could be a relevant key to understand the genetic architecture and gene networks underlying this complex trait. Our findings would completely fit with the additive model of inheritance previously proposed for HSCR in which the expression of the disease seems to depend on the contribution of different combinations of gene alleles acting in an additive or multiplicative fashion [3]. Regarding the variant M139I contained in the EGF domain, which is needed and sufficient to induce the receptor activation [19], it induces a significant reduction of the total NRG1 protein levels. Based on previous reports [8], we would suggest that this alteration could modify the receptor activation, which means functionality of the normal NRG1 protein. Regarding the R438H, it is known that the cytoplasmic domain regulates the proteolytic release of its extracellular domain in a sequence specific manner [38], [39]. This fact, together with the reported variant also located at this domain [8], lead us to speculate that R438H would affect the proteolytic cleavage of the pro-NRG1 protein.

In addition, the NRG1 receptor ErbB3, which is essential for neural crest cell survival, is regulated by Sox10 [29], [40], [41]. Therefore, as previously hypothesized [8] it would be plausible to speculate that reduced levels of NRG1 protein, observed in all three new variants, would affect the Sox10-mediated maintenance of ENS progenitors and contribute to the aganglionosis associated to HSCR disease.

In summary, the above mentioned results constitute the first report of *NRG1* mutations related to HSCR patients in a Caucasian population. These results are in accordance with the additive model previously proposed for HSCR [3], so we could hypothesize that these three novel variants, M111T, M139I and R438H would have functional consequences during embryonic development, probably being causing mutations which contribute to HSCR.

The delineation of *NRG1* function holds great promise for our understanding of the molecular pathways underlying HSCR.
